# Initial Invasive or Conservative Strategy in Heart Failure With Preserved Ejection Fraction and Coronary Artery Disease

**DOI:** 10.3389/fcvm.2022.822248

**Published:** 2022-03-18

**Authors:** Jun Gu, Jian-an Pan, Jun-feng Zhang, Chang-qian Wang

**Affiliations:** Department of Cardiology, Shanghai Ninth People’s Hospital, Shanghai Jiao Tong University School of Medicine, Shanghai, China

**Keywords:** heart failure with preserved ejection fraction, coronary artery disease, revascularization, propensity score matching, outcomes

## Abstract

**Background:**

In patients with both heart failure with preserved ejection fraction (HFpEF) and coronary artery disease (CAD), whether adopting an initial invasive strategy benefits better in clinical outcomes compared with those who received an initial conservative strategy remains inconclusive.

**Methods:**

With data from the heart failure (HF) cohort study, we analyzed patients who had HFpEF and CAD amenable to the invasive intervention using propensity score matching of 1:1 ratio to compare the initial invasive strategy and the initial conservative strategy of medical therapy alone. The primary outcome was the composite endpoints of all-cause mortality or cardiovascular hospitalization, and the secondary outcome was all-cause mortality or cardiovascular hospitalization.

**Results:**

Of 1,718 patients, 706 were treated with the invasive strategy and 1,012 with the conservative strategy initially. Propensity score matching was used to assemble a matched cohort of 1,320 patients receiving the invasive intervention (660 patients) or the medical therapy alone (660 patients). With a follow-up of 5 years, 378 (57.3%) in the invasive-strategy group and 403 (61.1%) in the conservative-strategy group reached the primary endpoint, and there was no significant difference in the rate of the primary endpoint (*P* = 0.162). The initial invasive strategy only improved the secondary outcome of cardiovascular hospitalization (*P* = 0.035). Also, the multivariable Cox regression model revealed that antiplatelet therapy, angiotensin-converting enzyme inhibitor/angiotensin II receptor blocker (ACEI/ARB), or statin prescription was associated with a decreased risk of the primary outcome.

**Conclusion:**

In this well-profiled, propensity-matched cohort of patients with HFpEF and CAD, the addition of invasive intervention to medical therapy did not improve the long-term composite of all-cause mortality or cardiovascular hospitalization.

## Introduction

Heart failure (HF) is generally considered a substantial source of morbidity and mortality globally, given an estimation of affecting 1–2% of the general population in western countries with more than 6 million adults in the United States alone (1). Approximately one-half of HF patients are suffering from HF with preserved ejection fraction (HFpEF) (2). Despite advances in the treatment of HFpEF consistently occur, outcomes of these patients are still not optimistic, and thus, the urgent need to identify and treat on specific target is proposed (2). Regarding etiologies, reversible ischemia and infarction related to coronary artery disease (CAD) remain the most common contributions of HFpEF (3–5). Also, revascularization is often advocated to improve the ventricular function and prognosis of HF patients caused by CAD, especially when there is evidence of extensive myocardial survival (6). However, no randomized trials of revascularization treatment vs. medical therapy have yet been conducted in HFpEF, and there are limited observational data on the prevalence and correlation of percutaneous coronary intervention (PCI) in HFpEF patients (7). The role of initial invasive strategy (angiography and revascularization when feasible) in addition to medical therapy in the management of HFpEF and CAD remains unclear.

We used data from our HF cohort study to explore the impact of an initial invasive strategy in addition to medical therapy on outcomes for patients with HFpEF and CAD, and the composite endpoints of death or cardiovascular hospitalization were compared.

## Materials and Methods

### Design and Setting

The patients in this study were extracted from our longitudinal HF cohort at Shanghai Ninth People’s Hospital, Shanghai Jiao Tong University School of Medicine, and a series of studies on the etiology and prognosis of HF have been carried out in recent years (8–11). To conduct this study, we retrospectively enrolled subjects with HFpEF and symptomatic CAD. The diagnosis of CAD included positive stress test, history of angina with ischemic change on electrocardiogram (ECG), previous myocardial infarction (MI) attack, or angina with obvious stenosis lesion in coronary computed tomography angiography (CCTA). HFpEF was defined by clinical features of HF with left ventricular ejection fraction (LVEF) greater than or equal to 50%, which was based on the 2021 ESC-HF guideline (2), and the natriuretic peptide and echocardiographic demonstration of structural and/or functional changes of the heart were the prerequisites for the diagnosis of HFpEF. Recruitment occurred either where the patient was in the hospital for a primary diagnosis of HFpEF (i.e., the assessment was performed following the stabilization of the acute HF) or in the outpatient setting within 3 months of an episode of decompensated HF (requiring hospitalization or treatment in an outpatient setting). Exclusion criteria were defined as follows: end-stage renal failure (estimated glomerular filtration rate (eGFR) < 30 ml/min/1.73 m^2^) or severe liver disease; hypertrophic cardiomyopathy or infiltrative cardiomyopathy; valvular heart disease; congenital heart disease; prior coronary artery bypass graft (CABG) surgery; recent acute coronary syndrome (≤90 days prior to enrollment); and any serious non-cardiovascular disease with a life expectancy of 12 months or less. All procedures were conducted under the guidance of the Declaration of Helsinki and were approved by the Ethics Committee and Independent Review Board of Shanghai Ninth People’s Hospital, Shanghai Jiao Tong University School of Medicine (SH9H-2019-T160-2), and informed consent was obtained from all patients.

### Treatment Strategies

The choice of treatment regimen (initial invasive or conservative strategy) was determined by the doctors in charge and the patient’s treatment willingness. The initial invasive strategy consisted of medical therapy, angiography, and revascularization when feasible, while the initial conservative strategy included medical therapy alone, with angiography reserved for failure of medical therapy (recurrent ischemic episodes, hemodynamic instability, overt congestive HF, or serious ventricular arrhythmias despite adequate medical therapy). Coronary angiography and revascularization procedures were conducted using standard techniques. The revascularization procedures, such as thrombectomy, pre-dilatation, stenting, and/or post-dilatation, were performed at the discretion of each operator. Decisions about the type of revascularization (PCI or CABG) were deferred to the local heart team. Uses of antiplatelet therapy, β-blockers, statins, and angiotensin-converting enzyme inhibitor/angiotensin II receptor blocker (ACEI/ARB) were followed the HF and CAD guidelines.

Propensity score matching was performed to avoid selection biases resulting from the non-random assignment in this observational study. The following clinically relevant baseline variables included in the matching process were age, gender, body mass index (BMI), dyslipidemia, hypertension, diabetes, smoking, history of MI, stroke, chronic obstructive pulmonary disease (COPD), atrial fibrillation, New York Heart Association (NYHA) functional class, hazard ratio (HR), systolic blood pressure (SBP), diastolic blood pressure (DBP), eGFR, hemoglobin, B-type natriuretic peptide (BNP), LVEF, left atrium diameter (LAD), E/e’, and medications. The initial invasive group was matched at a 1:1 ratio to the conservative group.

### Clinical Follow-Up and Endpoints

The follow-up started from the first day of the treatment strategy. The primary outcome was the composite of all-cause mortality or cardiovascular hospitalization. The secondary outcome was all-cause mortality or cardiovascular hospitalization. Most of the patients visited our outpatient clinic at least every 3 months. While if the patients did not appear at their scheduled clinic, they were interviewed by telephone annually. Information regarding the primary or secondary outcomes was documented in chart records and *via* telephonic interviews.

### Statistical Analysis

The SPSS Statistical Software version 22.0 (SPSS Inc., Chicago, IL, United States) was used for statistical analysis. Continuous variables were expressed as means ± standard deviations and compared with the unpaired two-sided Student’s *t*-test; categorical variables were expressed as frequency and percentage (%) and compared by chi-square test or Fisher’s exact test. Cox proportional hazards regression model was used to explore the association between risk factors and the risk of all-cause mortality or composite endpoints. All predictors with a significance of *P* ≤ 0.10 in the univariable analysis and forced inclusion variables that were considered as important predictors of clinical endpoints were entered into the multivariable model. HRs and corresponding 95% confidence intervals (CIs) were reported. The Kaplan–Meier method was used to estimate the Event-free survival curves with significance based on the log-rank test. *P*-value less than 0.05 (two-tailed) was considered statistically significant.

## Results

### Baseline Characteristics and Medical Therapy

**FIGURE 1:**
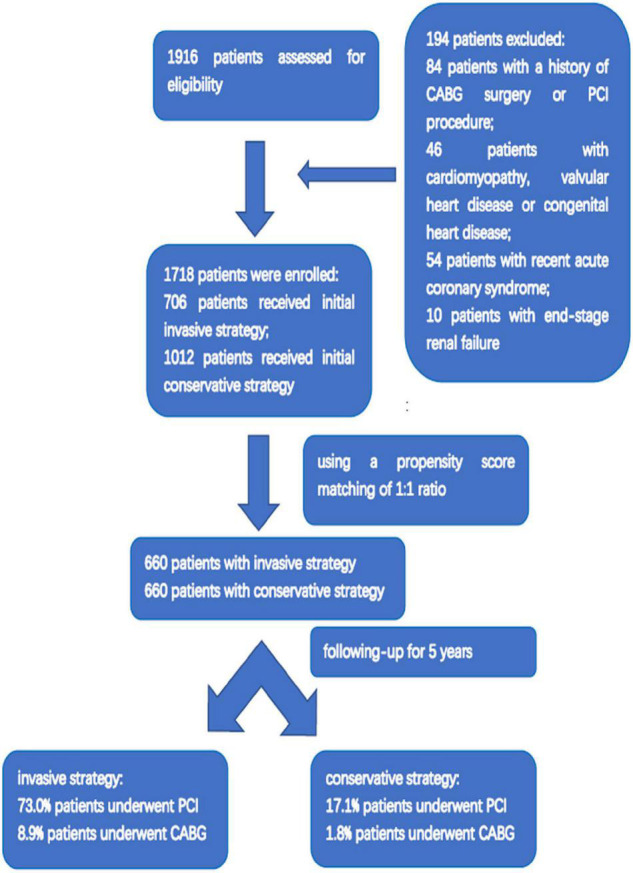
Flowchart of the study protocol.

From 1 January 2007 to 31 December 2015, a total of 1,916 patients with HFpEF and CAD were included, in which 796 patients had an initial invasive strategy, while 1,120 patients received initial conservative treatment. Based on the inclusion and exclusion criteria, the final study population comprised 706 patients with an invasive strategy and 1,012 patients received conservative treatment. After being matched with propensity score, a total of 660 patients receiving an invasive strategy and 660 patients receiving a conservative treatment were finally enrolled (Figure 1).

Table 1 demonstrated the demographic and baseline characteristics of the two groups. Compared with patients receiving a conservative therapy, those patients with an invasive intervention were younger, more likely to have dyslipidemia and a higher level of BNP, and a trend for better renal function. Besides, patients in the invasive intervention group received more proportions of CAD standard therapy, such as antiplatelet treatment, ACEI/ARB, beta-blockers, and statin prescription. After propensity score matching, these two treatment groups were well balanced for baseline demographic, comorbidities, clinical and echocardiographic characteristics, and medications (Table 2). As for another important factor for HFpEF and CAD patients, we calculated the cardiovascular hospitalization within 1 year prior to treatment strategy, and no significant difference was found before (33.0% vs. 35.0%, *P* = 0.255) and after (32.0% vs. 34.7%, *P* = 0.293) propensity score matching. Besides, the characteristics of the patients who could not be matched in the invasive group were described in Supplementary Table 1.

**TABLE 1:** Baseline clinical characteristics and medications.

Parameter	Total *n* = 1718	Conservative *n* = 1012	Invasive *n* = 706	*P* value	ASD
**Demographic characteristics**					
Age, years	70.4 ± 6.5	70.9 ± 6.5	69.7 ± 6.4	<0.001	0.182
Gender, female	769 (44.8)	461 (45.6)	308 (43.6)	0.429	0.039
BMI (kg/m^2^)	24.7 ± 2.1	24.8 ± 2.1	24.6 ± 2.1	0.132	0.074
**Cardiovascular risk factors**					
Dyslipidaemia	539 (31.4)	298 (29.4)	241 (34.1)	0.039	0.099
Hypertension	1239 (72.1)	720 (71.1)	510 (72.2)	0.927	0.005
Diabetes	559 (32.5)	329 (32.5)	220 (31.2)	0.309	0.050
Smoking	573 (33.4)	336 (33.2)	237 (33.6)	0.874	0.008
**Medical history**					
History of MI	240 (14.0)	149 (14.7)	91 (12.9)	0.281	0.055
Stroke	186 (10.8)	109 (10.8)	77 (10.9)	0.929	0.004
COPD	177 (10.3)	99 (9.8)	78 (11.0)	0.396	0.040
Atrial fibrillation	534 (31.1)	317 (31.3)	217 (30.7)	0.796	0.013
**Ardiac parameters**					
NYHA class, I/II/III/IV	152/642/816/608	88/380/480/64	64/262/336/44	0.993	0.001
Heart rate, bpm	79.0 ± 8.2	78.9 ± 8.6	79.0 ± 7.7	0.742	0.017
SBP, mmHg	131.0 ± 11.5	131.3 ± 11.6	130.5 ± 11.5	0.152	0.072
DBP, mmHg	77.5 ± 8.2	77.7 ± 8.7	77.2 ± 7.5	0.237	0.064
**Laboratory variables**					
eGFR (mL/min/1.73 m^2^)	60.7 ± 8.8	60.4 ± 8.4	61.2 ± 9.3	0.076	0.082
Hemoglobin (g/dL)	117.2 ± 14.2	117.2 ± 14.1	117.4 ± 14.5	0.877	0.007
BNP (pg/mL)	762.8 ± 266.2	774.7 ± 277.3	745.7 ± 248.6	0.026	0.117
**Medications**					
Anti-platelet	1383 (80.5)	759 (75.0)	624 (88.4)	<0.001	0.154
Anti-coagulation	169 (9.8)	100 (9.9)	69 (9.8)	0.941	0.004
ACEI/ARB	1203 (70.0)	684 (67.6)	519 (73.5)	0.008	0.134
Beta-blocker	1052 (61.2)	581 (57.4)	471 (66.7)	<0.001	0.197
Statin	1328 (77.3)	716 (70.8)	612 (86.7)	<0.001	0.298
Spironolactone	438 (25.5)	256 (25.3)	182 (25.8)	0.821	0.028
**Echo data**					
LVEF (%)	58.7 ± 4.6	58.6 ± 4.6	58.9 ± 4.5	0.277	0.055
LAD (mm)	42.7 ± 3.6	42.8 ± 3.5	42.5 ± 3.6	0.210	0.061
E/e’	13.7 ± 1.8	13.7 ± 1.8	13.6 ± 1.8	0.093	0.083

*Data are expressed as mean ± SD, or n (%).*

*ASD, absolute standardized difference (ASD); BMI, body mass index; MI, myocardial infarction; COPD, chronic obstructive pulmonary disease; NYHA, New York Heart Association functional class; SBP, systolic blood pressure; DBP, diastolic blood pressure; eGFR, estimated glomerular filtration rate; BNP, B-type natriuretic peptide; ACEI/ARB, angiotensin-converting enzyme inhibitor/angiotensin II receptor blocker; LVEF, left ventricular ejection fraction; LAD, left atrium diameter; E/e’, mitral Doppler early velocity/mitral annular early velocity.*

**TABLE 2:** Baseline clinical characteristics and medications after propensity score matching.

Parameter	Total *n* = 1320	Conservative *n* = 660	Invasive *n* = 660	*P* value	ASD
**Demographic characteristics**					
Age, years	69.8 ± 6.3	69.6 ± 6.3	69.9 ± 6.4	0.479	0.020
Gender, male	586 (44.4)	292 (44.2)	294 (44.5)	0.912	0.018
BMI (kg/m^2^)	24.7 ± 2.1	24.8 ± 2.1	24.7 ± 2.1	0.331	0.036
**Cardiovascular risk factors**					
Dyslipidaemia	433 (32.8)	216 (32.7)	217 (32.9)	0.953	0.003
Hypertension	935 (70.8)	480 (72.7)	485 (73.5)	0.130	0.032
Diabetes	429 (32.5)	214 (32.4)	215 (32.6)	0.953	0.022
Smoking	447 (33.9)	220 (33.3)	227 (34.4)	0.684	0.009
**Medical history**					
History of MI	192 (14.5)	88 (13.3)	84 (12.7)	0.744	0.052
Stroke	151 (11.4)	73 (11.1)	68 (10.3)	0.656	<0.001
COPD	136 (10.3)	67 (10.2)	69 (10.5)	0.856	0.028
Atrial fibrillation	405 (30.7)	205 (31.1)	200 (30.3)	0.765	0.041
**Cardiac parameters**					
NYHA class, I/II/III/IV	120/496/632/72	59/245/319/37	61/251/313/35	0.975	0.023
Heart rate, bpm	78.9 ± 8.2	79.0 ± 7.7	78.7 ± 8.7	0.321	0.005
SBP, mmHg	130.3 ± 11.1	130.5 ± 11.3	130.2 ± 11.0	0.603	0.021
DBP, mmHg	78.9 ± 8.2	77.3 ± 7.5	77.2 ± 8.5	0.754	0.050
**Laboratory variables**					
eGFR (mL/min/1.73 m^2^)	61.0 ± 8.7	60.9 ± 9.3	61.2 ± 8.0	0.523	0.054
Hemoglobin (g/dL)	117.5 ± 14.5	117.1 ± 15.0	117.9 ± 13.9	0.371	0.020
BNP (pg/mL)	763.4 ± 259.8	752.9 ± 252.4	773.9 ± 266.7	0.142	0.012
**Medications**					
Anti-platelet	1150 (87.1)	565 (85.6)	585 (88.6)	0.100	0.006
Anti-coagulation	127 (9.6)	63 (9.5)	64 (9.7)	0.926	0.015
ACEI/ARB	981 (74.3)	479 (72.6)	502 (76.1)	0.147	0.013
Beta-blocker	872 (66.1)	434 (65.8)	438 (66.4)	0.816	0.031
Statin	1118 ()	541 (82.0)	577 (87.4)	0.006	0.055
Spironolactone	350 (26.5)	170 (25.8)	181 (27.4)	0.493	0.039
**Echo data**					
LVEF (%)	58.8 ± 4.6	58.8 ± 4.4	58.8 ± 4.7	0.966	0.020
LAD (mm)	42.6 ± 3.7	42.6 ± 3.7	42.57 ± 3.7	0.741	0.034
E/e’	13.6 ± 1.8	13.6 ± 1.8	13.6 ± 1.7	0.804	0.019

*Data are expressed as mean ± SD, or n (%).*

*ASD, absolute standardized difference (ASD); BMI, body mass index; MI, myocardial infarction; COPD, chronic obstructive pulmonary disease; NYHA, New York Heart Association functional class; SBP, systolic blood pressure; DBP, diastolic blood pressure; eGFR, estimated glomerular filtration rate; BNP, B-type natriuretic peptide; ACEI/ARB, angiotensin-converting enzyme inhibitor/angiotensin II receptor blocker; LVEF, left ventricular ejection fraction; LAD, left atrium diameter; E/e’, mitral Doppler early velocity/mitral annular early velocity.*

### Evaluation for Ischemia

More than one-half of HFpEF patients underwent stress testing. Treadmill ECG testing was performed in 28%, stress echocardiography in 9%, nuclear testing in 23%, and cardiovascular magnetic resonance in 3.4%. Of note, 52% of enrolled patients underwent CCTA to evaluate the lesions of coronary disease.

### Use of Invasive Procedure

Of all patients in the invasive-strategy group, 100% underwent angiography and 82% underwent revascularization (PCI in 89% and CABG in 11%). In the conservative-strategy group, 22% of the patients underwent angiography and 15% underwent revascularization (PCI in 88% and CABG in 12%). A calculated number of 296 patients within the conservative-strategy group received invasive procedures including repeated ones, and that number in the invasive-strategy group reached 1,452. For revascularization, 347 of a total 541 patients in the invasive-strategy group received complete revascularization while partial revascularizations were conducted on the other 194. In the conservative group, 125 patients were conducted with the revascularization of which 67 were complete and 58 were partial. Angiographic characteristics and procedural data of patients in both groups were provided in Table 3.

**TABLE 3:** Angiographic characteristics and revascularization procedures.

	Invasive group *n* = 660	Conservative group *n* = 145
**Number of native vessel with ≧50% stenosis (QCA)**		
0	32 (4.8)	4 (2.8)
1	143 (21.7)	33 (22.8)
2	241 (36.5)	46 (31.7)
3	244 (37.0)	62 (42.8)
**Native vessel with ≧50% stenosis (QCA)**		
LM	21 (3.2)	3 (2.1)
LAD	475 (72.0)	108 (74.5)
LCX	436 (66.1)	88 (60.7)
RCA	431 (65.3)	68 (46.9)
PCI	482 (73.0)	113 (77.9)
CABG	59 (8.9)	12 (8.3)

*Data are expressed as mean ± SD, or n (%).QCA, quantitative coronary angiography; LM, left main;LAD, left anterior descending; LCX, left circumflex; RCA, right coronary artery; PCI, percutaneous coronary intervention; CABG, coronary artery bypass graft.*

### Primary Outcome

**FIGURE 2:**
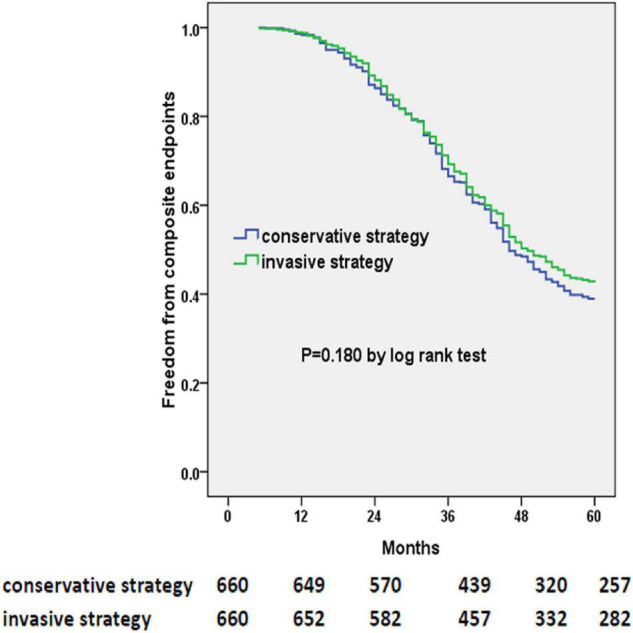
Kaplan–Meier curves of freedom from composite endpoints. The numbers at the bottom of the figure are “number at risk.”

The primary outcome occurred in 378 patients of the invasive-strategy group and in 403 patients of the conservative-strategy group (*P* = 0.162). At 5 years, in prespecified covariate-adjusted Cox model analysis, the estimated HR with the invasive strategy as compared with the conservative strategy was 0.900 (95% CI: 0.781 to 1.037; *P* = 0.145). Also, the multivariable Cox regression model (Table 4) revealed that older age, previous MI, higher BNP level, or NYHA functional class was associated with an increased risk of composite endpoints, while antiplatelet therapy, ACEI/ARB, or statin prescription was associated with a decreased risk of composite endpoints (i.e., the univariate model is shown in Supplementary Table 2). The Kaplan–Meier plot for the occurrence of composite endpoints between two strategies is presented in Figure 2, in which we did not find evidence of a significant difference in the 5-year event-free time (*P* = 0.180).

**TABLE 4:** Multivariable cox analysis for composite endpoints.

	HR	95% CI	*P* value
Age	1.013	1.001–1.024	0.033
eGFR	0.993	0.984–1.001	0.084
Prior MI	1.300	1.068–1.582	0.009
Arial fibrillation	1.305	1.123–1.518	0.001
Diabetes	1.055	0.908–1.225	0.487
Hypertension	1.081	0.923–1.268	0.334
COPD	0.852	0.668–1.086	0.196
BNP tertile	1.163	1.062–1.271	0.001
NYHA class	1.121	1.017–1.234	0.021
ACEI/ARB	0.811	0.732–0.987	0.009
Betablocker	0.904	0.779–1.048	0.181
Statin	0.850	10.811–2.779	0.033
Antiplatelet therapy	0.817	0.702–0.951	0.009
LAD	1.007	0.988–1.026	0.492
E/e’	1.034	0.994–1.076	0.100
LVEF	0.995	0.980–1.011	0.561
Invasive strategy	0.900	0.781–1.037	0.145
Complete revascularization	0.896	0.762–1.045	0.086

*eGFR, estimated glomerular filtration rate; MI, myocardial infarction; COPD, chronic obstructive pulmonary disease; B-type natriuretic peptide; NYHA, New York Heart Association functional class; ACEI/ARB, angiotensin-converting enzyme inhibitor/angiotensin II receptor blocker; LAD, left atrium diameter; E/e’, mitral Doppler early velocity/mitral annular early velocity; LVEF, left ventricular ejection fraction.*

### Secondary Outcomes

**FIGURE 3:**
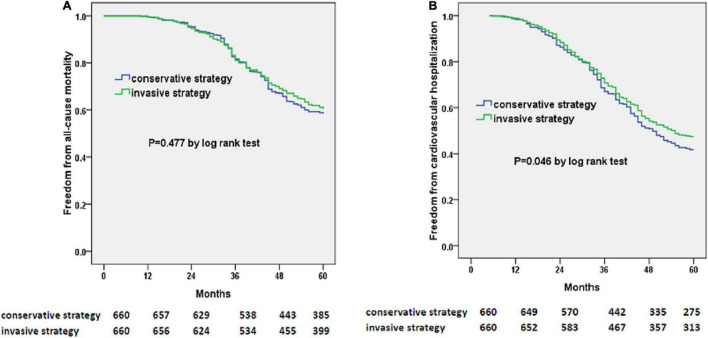
Kaplan–Meier curves of freedom from all-cause mortality **(A)** and cardiovascular hospitalization **(B)**. The numbers at the bottom of the figure are “number at risk.”

At 5 years, there were 261 deaths in the invasive-strategy group and 275 deaths in the conservative-strategy group (*P* = 0.433). Also, more patients within the conservative-strategy group were hospitalized for cardiovascular reasons (385/660 vs. 347/660, *P* = 0.035). The Kaplan–Meier plots showed a decreased risk of cardiovascular hospitalization instead of all-cause mortality in the invasive-strategy group in Figure 3.

## Discussion

In this well-profiled, propensity-matched cohort of patients with HFpEF and CAD, there were no differences regarding the composite endpoints of all-cause mortality or cardiovascular hospitalization between patients treated with an initial invasive therapy compared with an initial medical treatment alone during the 5-year follow-up.

Notably, HFpEF is considered to be a heterogeneous syndrome, which is driven by a series of comorbidities. Due to the diversity of patients, ACEI/ARB, β-blockers, and spironolactone have failed to achieve clinical efficacy in randomized controlled trials (6). Also, our previous study revealed that these three treatments have nothing to do with the improvement in the outcome of the HFpEF cohort (8). A recent study investigated the clinical, structural, functional, hemodynamic, and outcome characteristics in a rigorously phenotyped group of patients hospitalized for HFpEF (3). Among HF patients, 52.6% had reduced ejection fraction (EF), and 47.4% had preserved EF (3). In addition, studies have shown that the main subtypes of HF after acute MI are HF with midrange ejection fraction (HFmrEF) and HFpEF (4). Also, HFpEF patients with CAD exhibit a higher risk of all-cause mortality and sudden death compared with those without CAD (4). Ischemic heart disease (IHD) increases the risk of major adverse renal and cardiovascular events (MARCE) by approximately 20% in patients with HFpEF (4). Moreover, during the long-term follow-up, HFpEF patients with significant anatomical CAD have a higher mortality rate (3). Similarly, our previous study identified IHD as an independent risk factor for the deterioration of LVEF and clinical outcomes in patients with HFpEF (8). Therefore, the unmet need is recommended to create specific interventions for this subgroup of HFpEF (7).

Our latest research showed that patients with HFpEF and CAD might benefit from the long-term ACEI/ARB treatment (9). Another study also demonstrated the differences in medical therapy response among different HFpEF subgroups, for instance, inhospital beta-blocker treatment was significantly associated with a reduction in hospital mortality only in HFpEF patients with hypertension, whereas in hospital diuretic treatment was significantly associated with the better outcome only in HFpEF patients without hypertension (12). The recent PARAGON-HF trial compared the effects of sacubitril-valsartan and only valsartan in HFpEF patients with a composite of HF hospitalization and cardiovascular death as the primary outcome (13–15). Although treatment with sacubitril-valsartan did not reduce the primary outcome significantly (*P* = 0.059), sub-analyses still suggested beneficial effects in female patients and those with an LVEF between 45% and 57%. In this study, we found that long-term prescriptions of antiplatelet, statin, and ACEI/ARB were associated with better clinical prognosis in patients with HFpEF and CAD.

Whether PCI performed for acute or chronic HF during hospitalization improves the prognosis of patients with reduced LVEF remains questionable. A meta-analysis showed that revascularization strategies were superior to medical therapy in improving the survival rate in patients with IHD and reduced LVEF (16). However, in another well-profiled, propensity-matched cohort of patients with stable CAD amenable to PCI and moderate or severe left ventricular systolic dysfunction (LVSD), the addition of PCI to medical therapy did not improve long-term mortality or the composite of mortality or cardiovascular hospitalization (17). A conservative management strategy may not be inferior to one of coronary arteriography that aimed to revascularize in patients with HF, LVSD, and extensive myocardial viability (18). Currently, no clear recommendations or guidelines regarding the role of PCI in the management of HF with reduced EF (HFrEF) have been proposed due to the lack of evidence from randomized controlled trials. Some believed that patients with complex CAD and LVSD might have reverse LV remodeling after high-risk PCI, and this was related to improved outcomes (19, 20). In REVIVED-BCIS2 (21), a currently ongoing prospective, multicenter, open-label, randomized, and controlled trial that enrolled a total of 700 patients with the inclusion criteria of LVEF < 35% and extensive coronary disease with myocardial viability in at least 4 dysfunctional segments that can be percutaneously revascularized and also a strategy of PCI with optimal medical therapy (OMT) vs. OMT alone is being investigated. The primary endpoint in this trial refers to the all-cause death or hospitalization for HF after a minimum follow-up of 24 months. The results of this trial involving the first randomized data on the efficacy and safety of PCI in patients with HFrEF were wildly expected.

With regard to patients with HFpEF, there has been also no randomized controlled trial to assess the impact of PCI in patients with acute and chronic HFpEF. The observational data about the prevalence and associations of PCI in HFpEF patients were also limited (7). A meta-analysis of both acute and chronic HFpEF patients demonstrated a pooled CAD prevalence of 47% (22). Another study showed that 80% of patients with acute HFpEF accompanied by obvious CAD received coronary revascularization, and 63% of them received PCI (3). Complete revascularization was associated with improved prognosis in patients with acute and chronic HFpEF (3, 23). In the CHART-2 study of adults with chronic HF, residual stenosis after PCI was independently associated with higher mortality rates in patients with LVEF ≥ 50% and LVEF = 40–49% groups but not in patients with LVEF < 40%(23). Similarly, complete revascularization in patients with acute HFpEF has been associated with significantly higher rates of survival and less LVEF decline compared with patients who did not undergo complete revascularization (3). In our present propensity-matched cohort study of patients suffering both HFpEF and CAD when treated with initial invasive therapy, an improved outcome exists only in terms of cardiovascular hospitalization but not the primary outcome of all-cause mortality or cardiovascular hospitalization or secondary outcome of all-cause mortality. This suggests that the efficacy of revascularization therapy in this population needs to be further clarified. The reduction in the rate of cardiovascular hospitalization in the invasive group might be related to rescue invasive intervention due to the medical treatment fails in the conservative group. Besides, our study suggested that complete revascularization only exerted a decreasing trend for the primary composite, while the result did not reach a statistical difference (multivariable Cox analysis).

There were still some unavoidable limitations in our research. First of all, the results of an observational analysis of single-center data were not accurate enough. Although propensity score adjustments have been made, there were still other potential variables that might affect the results of the study. Therefore, a sufficiently powerful randomized clinical trial is needed for further proof. Second, during the research period, the PCI technology of CAD and the drug treatment of HFpEF have been significantly improved, such as angiotensin receptor neprilysin inhibitor (ARNI) (13–15) and sodium-dependent glucose transporter 2 inhibition agent (SGLT2i) (24) for HFpEF. However, no participants in our study prescribed ARNI, and only a very small number of patients received SGLT2i prescriptions. Finally, although more than one-half of HFpEF patients underwent stress testing and 52% of enrolled patients underwent CCTA, the lack of invasive assessment of coronary artery in each enrolled patient is a deficiency of this study.

## Conclusion

When investigating the long-term results of initial conservative treatment or invasive intervention in patients with HFpEF and CAD, we found that there was no significant difference in the composite endpoints of all-cause mortality or cardiovascular hospitalization. As for the secondary endpoints, the initial invasive strategy was associated with similar risks of all-cause mortality but reduced the risk of cardiovascular hospitalization. Besides, standard medical therapy for CAD was also associated with greater freedom from composite endpoints.

## Data Availability Statement

The raw data supporting the conclusions of this article will be made available by the authors, without undue reservation.

## Ethics Statement

The studies involving human participants were reviewed and approved by the Ethics Committee and Independent Review Board Shanghai Ninth People’s Hospital. The patients/participants provided their written informed consent to participate in this study.

## Author Contributions

JG designed the research. J-AP collected the data. JG and J-FZ analyzed the data. JG and C-QW wrote the manuscript. All authors contributed to the article and approved the submitted version.

## Conflict of Interest

The authors declare that the research was conducted in the absence of any commercial or financial relationships that could be construed as a potential conflict of interest.

## Publisher’s Note

All claims expressed in this article are solely those of the authors and do not necessarily represent those of their affiliated organizations, or those of the publisher, the editors and the reviewers. Any product that may be evaluated in this article, or claim that may be made by its manufacturer, is not guaranteed or endorsed by the publisher.
