# Supplementary material for: Initial Invasive or Conservative Strategy in Heart Failure With Preserved Ejection Fraction and Coronary Artery Disease
Source: Front Cardiovasc Med. 2022 Mar 18;9:822248. doi: 10.3389/fcvm.2022.822248 (PMC8971278; doi:10.3389/fcvm.2022.822248)
Supplement: Supplementary file 1 [file Table_1.docx]

**Table S1 Baseline clinical characteristics and medications for PSM and non-PSM patients in invasive group**

| parameter | total  n=706 | PSM  n=660 | Non-PSM  n=46 | P value |
| --- | --- | --- | --- | --- |
| **demographic characteristics** |  |  |  |  |
| age, years | 69.7±6.4 | 69.9±6.4 | 66.8±7.1 | 0.001 |
| gender, male | 308(43.6) | 294(44.5) | 14(30.4) | 0.062 |
| BMI (kg/m^2^) | 24.6±2.1 | 24.7±2.1 | 24.1±2.0 | 0.061 |
| **cardiovascular risk factors** |  |  |  |  |
| dyslipidaemia | 241(34.1) | 217(32.9) | 24(52.2) | 0.008 |
| hypertension | 510(72.2) | 485(73.5) | 25(54.3) | 0.005 |
| diabetes | 220(31.2) | 215(32.6) | 5(10.9) | 0.002 |
| smoking | 237(33.6) | 227(34.4) | 10(21.7) | 0.079 |
| **medical history** |  |  |  |  |
| history of MI | 91(12.9) | 84(12.7) | 7(15.2) | 0.626 |
| stroke | 77(10.9) | 68(10.3) | 9(19.6) | 0.051 |
| COPD | 78(11.0) | 69(10.5) | 9(19.6) | 0.057 |
| atrial fibrillation | 217(30.7) | 200(30.3) | 17(37.0) | 0.344 |
| **cardiac parameters** |  |  |  |  |
| NYHA class, I/II/III/IV | 64/262/336/44 | 61/251/313/35 | 3/11/23/9 | 0.001 |
| heart rate, bpm | 79.0±7.7 | 78.7±8.7 | 79.9±7.2 | 0.361 |
| SBP, mmHg | 130.5±11.5 | 130.2±11.0 | 129.7±10.8 | 0.622 |
| DBP, mmHg | 77.2±7.5 | 77.2±8.5 | 76.1±6.4 | 0.390 |
| **laboratory variables** |  |  |  |  |
| eGFR (mL/min/1.73 m^2^) | 61.2±9.3 | 61.2±8.0 | 62.0±7.2 | 0.510 |
| haemoglobin (g/dL) | 117.4±14.5 | 117.9±13.9 | 117.2±13.6 | 0.741 |
| BNP (pg/mL) | 745.7±248.6 | 773.9±266.7 | 636.6±166.2 | 0.002 |
| **medications** |  |  |  |  |
| anti-platelet | 624(88.4) | 585(88.6) | 39(84.8) | 0.430 |
| anti-coagulation | 69(9.8) | 64(9.7) | 5(10.9) | 0.796 |
| ACEI/ARB | 519(73.5) | 502(76.1) | 17(37.0) | <0.001 |
| beta-blocker | 471(66.7) | 438(66.4） | 33(71.7） | 0.454 |
| statin | 612(86.7) | 577(87.4) | 35(76.1) | 0.029 |
| spironolactone | 182(25.8) | 181(27.4) | 1(2.2) | <0.001 |
| **echo data** |  |  |  |  |
| LVEF(%) | 58.9±4.5 | 58.8±4.7 | 59.7±4.2 | 0.207 |
| LAD (mm) | 42.5±3.6 | 42.6±3.7 | 41.6±2.9 | 0.064 |
| E/e’ | 13.6±1.8 | 13.6±1.7 | 13.3±1.9 | 0.251 |

Data are expressed as mean ± SD, or n (%).

PSM: propensity score matching; BMI: body mass index; MI: myocardial infarction; COPD: chronic obstructive pulmonary disease; NYHA: New York Heart Association functional class; SBP: systolic blood pressure; DBP: diastolic blood pressure; eGFR: estimated glomerular filtration rate; BNP: B-type natriuretic peptide; ACEI/ARB: angiotensin-converting enzyme inhibitor/angiotensin II receptor blocker; LVEF: left ventricular ejection fraction; LAD: left atrium diameter; E/e’: mitral Doppler early velocity/mitral annular early velocity.
