# Supplementary material for: Initial Invasive or Conservative Strategy in Heart Failure With Preserved Ejection Fraction and Coronary Artery Disease
Source: Front Cardiovasc Med. 2022 Mar 18;9:822248. doi: 10.3389/fcvm.2022.822248 (PMC8971278; doi:10.3389/fcvm.2022.822248)
Supplement: Supplementary file 2 [file Table_2.docx]

**Table S2 Univariate cox analysis for composite endpoints**

|  | HR | 95% CI | P value |
| --- | --- | --- | --- |
| age | 1.015 | 1.001-1.028 | 0.030 |
| eGFR | 0.992 | 0.984-1.000 | 0.054 |
| Prior MI | 1.251 | 1.031-1.517 | 0.023 |
| arial fibrillation | 1.249 | 1.077-1.448 | 0.003 |
| diabetes | 1.030 | 0.889-1.195 | 0.297 |
| hypertension | 1.016 | 0.871-1.186 | 0.495 |
| COPD | 0.845 | 0.664-1.075 | 0.169 |
| BNP tertile | 1.156 | 1.058-1.263 | 0.001 |
| NYHA class | 1.110 | 1.009-1.220 | 0.031 |
| ACEI/ARB | 0.811 | 0.694-0.947 | 0.008 |
| betablocker | 0.911 | 0.787-1.056 | 0.215 |
| statin | 0.858 | 0.740-0.995 | 0.043 |
| antiplatelet therapy | 0.838 | 0.720-0.974 | 0.021 |
| LAD | 1.005 | 0.986-1.024 | 0.606 |
| E/e’ | 1.036 | 0.996-1.077 | 0.080 |
| LVEF | 0.998 | 0.983-1.014 | 0.799 |
| invasive strategy | 0.909 | 0.790-1.047 | 0.185 |
| complete revascularization | 0.892 | 0.760-1.047 | 0.080 |

eGFR: estimated glomerular filtration rate; MI: myocardial infarction; COPD: chronic obstructive pulmonary disease; B-type natriuretic peptide; NYHA: New York Heart Association functional class; ACEI/ARB: angiotensin-converting enzyme inhibitor/angiotensin II receptor blocker; LAD: left atrium diameter; E/e’: mitral Doppler early velocity/mitral annular early velocity; LVEF: left ventricular ejection fraction.
